# The Lectin Pathway of the Complement System—Activation, Regulation, Disease Connections and Interplay with Other (Proteolytic) Systems

**DOI:** 10.3390/ijms25031566

**Published:** 2024-01-26

**Authors:** József Dobó, Andrea Kocsis, Bence Farkas, Flóra Demeter, László Cervenak, Péter Gál

**Affiliations:** 1 Institute of Molecular Life Sciences, HUN-REN Research Centre for Natural Sciences, Hungarian Research Network, 1117 Budapest, Hungary; dobo.jozsef@ttk.hu (J.D.); kocsis.andrea@ttk.hu (A.K.); farkas.bence@ttk.hu (B.F.); 2 Cell Biology and Cell Therapy Group, Research Laboratory, Department of Internal Medicine and Hematology, Semmelweis University, 1085 Budapest, Hungary; flora.demeter@gmail.com (F.D.); cervenak.laszlo@med.semmelweis-univ.hu (L.C.)

**Keywords:** complement lectin pathway, pattern recognition, protease, MASP, endothelial cells, ischemia reperfusion injury

## Abstract

The complement system is the other major proteolytic cascade in the blood of vertebrates besides the coagulation–fibrinolytic system. Among the three main activation routes of complement, the lectin pathway (LP) has been discovered the latest, and it is still the subject of intense research. Mannose-binding lectin (MBL), other collectins, and ficolins are collectively termed as the pattern recognition molecules (PRMs) of the LP, and they are responsible for targeting LP activation to molecular patterns, e.g., on bacteria. MBL-associated serine proteases (MASPs) are the effectors, while MBL-associated proteins (MAps) have regulatory functions. Two serine protease components, MASP-1 and MASP-2, trigger the LP activation, while the third component, MASP-3, is involved in the function of the alternative pathway (AP) of complement. Besides their functions within the complement system, certain LP components have secondary (“moonlighting”) functions, e.g., in embryonic development. They also contribute to blood coagulation, and some might have tumor suppressing roles. Uncontrolled complement activation can contribute to the progression of many diseases (e.g., stroke, kidney diseases, thrombotic complications, and COVID-19). In most cases, the lectin pathway has also been implicated. In this review, we summarize the history of the lectin pathway, introduce their components, describe its activation and regulation, its roles within the complement cascade, its connections to blood coagulation, and its direct cellular effects. Special emphasis is placed on disease connections and the non-canonical functions of LP components.

## 1. Introduction and Brief History of the Lectin Pathway

The complement system is an important effector arm of the innate immune system. It consists of about 40 protein components, both fluid-phase and cell-surface molecules. This remarkable molecular network carries the properties typical of the immune system in general: it can recognize, label, and eliminate pathogens and the dangerously altered host cells. The recognition function is mediated by pattern recognition molecules (PRMs) such as C1q, MBL, and ficolins. These PRMs typically contain globular domains responsible for binding to dangerous structures and collagen-like arms to which serine protease components bind. From an enzymatic point of view, the system can be considered a proteolytic cascade system, like the blood coagulation and the fibrinolytic systems. These proteolytic cascade systems in the blood are evolutionary and functionally closely related, and they form a single proteolytic network [1]. For practical and didactical reasons, this unified network can be divided into separate cascade systems, but understandably, there is a lot of cross-talk between these systems.

Most of the serine proteases of the complement system are present as zymogens in the circulation. When the PRMs bind to the target surface, the associated serine proteases become activated and initiate the complement cascade by cleaving and activating the subsequent components. Since one active protease can cleave numerous zymogen molecules, the proteolytic cascade carries an enormous amplification potential. As a result, the complement activation is tightly regulated by various inhibitors to avoid self-tissue damage. Indeed, if the complement activation happens in an uncontrolled manner, it can contribute to the development and progression of serious disease conditions [2].

**Figure 1:**
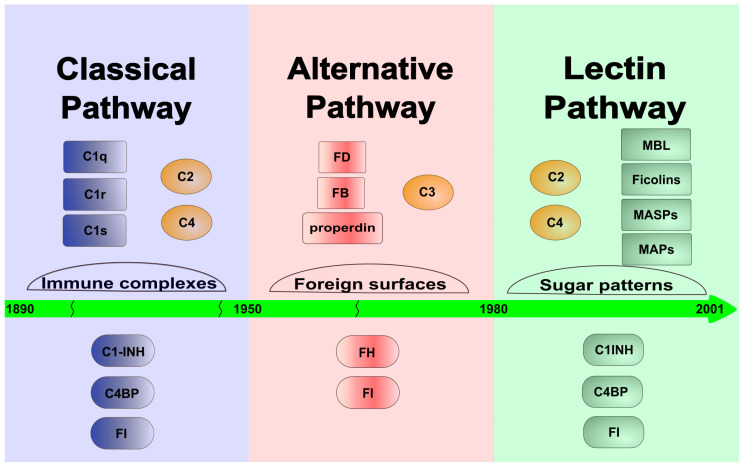
Timeline toward the discovery of the LP. The figure shows the time sequence of the discovery of the individual activation routes of the system. The classical pathway dominated the early 20th century, while the alternative pathway was described in the middle of that century. Finally, the lectin pathway was discovered and characterized at the end of the 20th and at the beginning of the 21st century. The figure shows the most important components (upper lane) and the most important fluid-phase inhibitors (lower lane) of each complement pathway.

There are three ways through which the complement system can activate: the classical, the alternative and the lectin pathways. The classical pathway was discovered first, at the turn of the 19th and 20th centuries, and it determined the general view of the complement system for a long time. At that time, the system was considered an effector mechanism of the adaptive immune response, which “complements” the action of the antibodies. Later, in the 1950s, an antibody-independent activation pathway, the alternative pathway, was discovered, but it became generally accepted only in the 1970s. The alternative pathway clearly linked the complement system to innate immunity and demonstrated that this molecular system can independently recognize and destroy the pathogens. The lectin pathway is the latest discovered activation route of the complement system (Figure 1). It was already observed in the 1980s that the MBL, in a complex with proteases, can activate the lectin pathway [3,4]. Initially, it was thought that MBL, like C1q of the classical pathway, binds C1r and C1s and initiates the classical pathway activation. Later, it turned out that MBL is associated with another protease called MASP (MBL-associated serine protease, earlier known as P100), which is able to cleave C4 and C2 to generate the C3 convertase complex [5]. A few years later, however, it turned out that “MASP” is a mixture of two proteases (MASP-1 and MASP-2), and the protease present in much smaller amounts (MASP-2) is responsible for cleaving C4 and C2 [6]. It was also shown that isolated MASP-2 can autoactivate in vitro and initiate the lectin pathway activation without the contribution of any other protease [7]. The conclusion drawn from this observation, i.e., that MASP-2 alone is sufficient for activation, defined the theory of lectin pathway activation for more than a decade. In 2001, a third protease component, MASP-3 was discovered, which is a product of the *MASP1* gene [8]. In the beginning, nothing was known about the biological role of MASP-3, and no physiological substrate was known either. For lack of a better theory, MASP-3 had been attributed an inhibitory role, despite the fact that it is an enzymatically active protease. Now, we know that MASP-3 plays a major role in complement activation and beyond. In the meantime, other, non-enzymatic components of the lectin pathway were also discovered. From both *MASP1* and *MASP2* genes, shorter N-terminal fragments lacking the catalytic domain (MAp19 and MAp44) are expressed via alternative splicing or polyadenylation [9,10]. The number of PRMs also has been increased by the discovery of ficolins (ficolin-1, -2 and -3) [11] and other collectins (CL-K1, CL-L1) [12].

The lectin pathway is perhaps the most complex activation pathway among the complement pathways; it has at least six PRMs, three serine proteases, and two non-catalytic fragments. Since the PRMs can have a different oligomerization status, from dimer to hexamer [13], and one PRM can bind different MASPs and MAps, many different activation complexes can form. We still do not have accurate data regarding the exact composition, concentration and distribution of these complexes in the blood. The lectin pathway represents one of the first lines of defense against invading pathogens. The activation of the lectin pathway initiates a complex innate immune response including cross-talks with other proteolytic cascade systems and direct cell activation by cleaving protease-activated receptors (e.g., endothelial cells). In this review, we summarize the present knowledge about the activation and regulation of the lectin pathway and the complex role of the lectin pathway in various physiological processes. We place particular emphasis on the role of the lectin pathway in the development of various diseases and on the possible therapeutic options. The non-canonical, non-immunological functions of certain lectin pathway components will also be discussed.

## 2. Genes and Components

**Figure 2:**
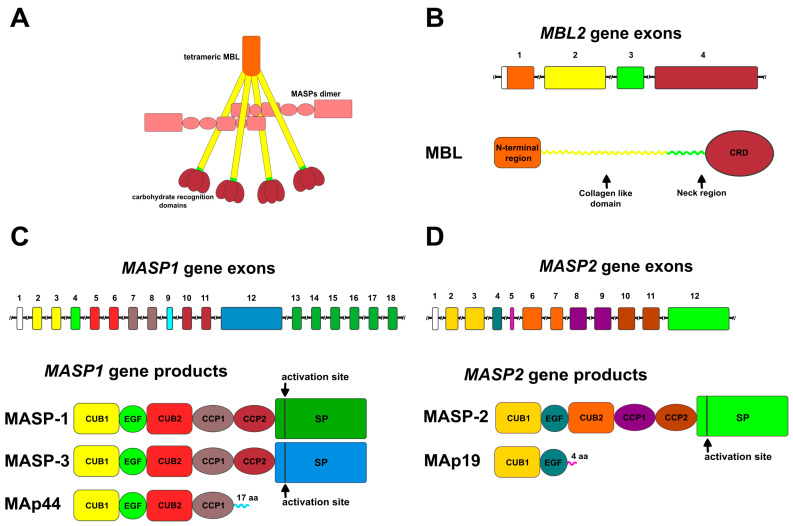
Genes and components of the LP. The figure shows a typical LP complex and the gene organization and the domain structure of the certain LP genes and LP proteins, respectively. The color codes of the polypeptide-encoding parts of the exons correspond to the color codes of the protein domains they encode in panels (**B**–**D**). The signal peptides (white) are removed from the mature proteins. (**A**) The schematic structure of a typical MBL/MASP complex. MBL is colored as in panel (**B**). (**B**) The *MBL2* gene encodes a single polypeptide chain. (**C**) The *MASP1* gene encodes three protein products. (**D**) The *MASP2* gene encodes two protein products. Abbreviation of the domain names: CUB: C1r/C1s, sea urchin Uegf, bone morphogenetic protein; EGF: epidermal growth factor; CCP: complement control protein; SP: serine protease; CRD: carbohydrate recognition domain; aa: amino acid (the number before aa indicates the length of the segment). Detailed description can be found in the text.

In this section, the primary focus will be on the components of the human lectin pathway (LP). The complement system of other mammals is typically very similar to the human complement system, and this stands for the lectin pathway as well. However, there might be slight differences. Some of the differences will be pointed out. As mentioned earlier, LP components can be divided into two classes of molecules, one being the pattern recognition molecules (PRMs) and the other being the class of MBL-associated serine proteases (MASPs) and proteins (MAps). MASPs and MAps form Ca^2+^-dependent dimers, and they are bound to the different PRMs also in a Ca^2+^-dependent fashion. A typical complex is shown in Figure 2A. As PRMs can be found in different oligomeric states, and higher oligomers can probably accommodate more than just one dimer of MASPs or MAps, Figure 2A depicts only the simplest case. Furthermore, MASPs and MAps might form heterodimers, which further complicates the picture [14,15]. All the activation complexes of the LP have heterogeneous composition, and they could be described as a mixture with varying stoichiometry. Below, components of the LP are described in more detail and also summarized in Figure 2.

### 2.1. Pattern Recognition Molecules (PRMs)

#### 2.1.1. Mannose-Binding Lectin (MBL)

The molecule after which the lectin pathway derived its name is mannose-binding lectin (MBL), previously also called mannan-binding lectin, mannan-binding protein (MBP), and P28/P32 of the Ra-reactive factor (Ra-RF) [5,16,17]. In humans, functional MBL is encoded by the *MBL2* gene, while *MBL1* is a pseudogene [18]. Interestingly, in mice and rats, both MBL genes are functional, and the resulting polypeptides are termed MBL-A and MBL-C [7]. The genetic and domain organization of human MBL is shown in Figure 2B. MBL is composed of four domains; an N-terminal Cys-rich domain, a collagen-like domain, a coiled-coil neck region, and a C-terminal carbohydrate recognition domain or C-type lectin domain. MBL, like all C-type lectins, requires Ca^2+^ for ligand binding. Being a lectin and also having a collagenous part, MBL is a typical member of the collectin protein family [19]. Three polypeptide chains form a protomer of about 76 kDa [20], as a triple helix is formed via the collagenous part and the neck region [21]. The protomers can further associate to form dimers, trimers, tetramers, and even higher oligomers via disulfide bonds between the Cys-rich domains of the individual chains. The major forms of human MBL appear to be the trimeric (3 × 3 chains) and the tetrameric (4 × 3 chains) forms [20].

#### 2.1.2. Other Collectins

Two soluble collectins have been described that are capable of activating the complement system along with MASPs [22,23,24]. These are collectin liver 1 (CL-L1) and collectin kidney 1 (CL-K1). CL-L1 is also called collectin 10, and it is encoded by the *COLLEC10* gene, while CL-K1 is also termed collectin 11, and it is encoded by the *COLLEC11* gene. The domain organization, oligomerization and structure of CL-L1 and CL-K1 are similar to those of MBL, but the two polypeptides have been shown to form heterocomplexes termed CL-LK [12,25], i.e. the individual chains of the oligomeric CL-LK molecules can be derived from either CL-L1 or CL-K1. A third collectin, CL-P1 (collectin placenta 1) or collectin 12, has been described, but it is mainly associated with the membranes and is not thought to be able to activate the LP [26].

#### 2.1.3. Ficolins

In humans, three ficolins are associated with MASPs and MAps, and all are shown to activate the complement cascade via the LP [27,28,29]. Ficolins are lectin-like molecules containing a fibrinogen-like domain at their C-terminus. They recognize acetyl groups in various compounds, including acetylated proteins [30,31], but their natural ligands are typically acetylated sugar moieties on the surface of bacteria. Hence, functionally, they are similar to true lectins. The fibrinogen-like domain has a Ca^2+^-binding site [32], but Ca^2+^ is not absolutely required for the ligand binding of ficolin-2 (L-ficolin) [30,33]. The fibrinogen-like domains of ficolins, unlike C-type lectins, seem to use multiple binding sites for ligands [32].

The three human ficolins are ficolin-1 (M-ficolin), ficolin-2 (L-ficolin, p35), and ficolin-3 (H-ficolin). Ficolin-3 (H-ficolin, also known as the Halso known asta antigen) has, by far, the highest serum concentration in humans (about 20 μg/mL) [34], and it is encoded by the *FCN3* gene. Ficolin-2 (L-ficolin), encoded by the *FCN2* gene, is the second most abundant in serum. Ficolin-1 (M-ficolin), encoded by the *FCN1* gene, has a very low serum concentration and is most associated with immune cells.

The structure of ficolins closely resembles that of the MBL. Three polypeptide chains form a homotrimer protomer, and the protomers form oligomers, but they lack the “neck” region. The degree of oligomerization of ficolins is thought to be between the tetrameric to octameric states [11,35,36]. On electron microscopic images, the typical oligomeric form of ficolins appears to be the tetrameric (4 × 3-chains) form [37]. The oligomers are held together by interchain disulfide bonds and/or noncovalent interactions at the N-terminal region. Interestingly, on non-reduced SDS-PA gels, H-ficolin appears as ladder of at least 12 bands composed of 1–12 or even more chains [38].

### 2.2. MBL-Associated Serine Proteases (MASPs) and Proteins (MAps)

#### 2.2.1. Products of the *MASP1* Gene

The *MASP1* gene encodes three known protein products, which are formed via alternative splicing or alternative polyadenylation [10]. The first discovered MBL-associated serine protease 1 (MASP-1) [5] is the main protease of the LP with a serum level of about 11 μg/mL [39]. MASP-1 was also called the P100 component of Ra-RF in mice [40]. MASP-3 was discovered later as a protease also associated with MBL isolated from human serum [8]. The two proteins share the same domain organization of six domains, and their sequence differs only in their serine protease (SP) domain (Figure 2C). The third splice product of the *MASP1* gene is MAp44 (also known as MAP-1), a short variant form of 44 kDa, which lacks an SP domain. Hence, it has an inhibitory role for the LP [10,41]. A fourth splice variant was detected at the mRNA level, but not at the protein level [42].

#### 2.2.2. Products of the *MASP2* Gene

The *MASP2* gene has two protein products, MASP-2 and MAp19 [9,43]. MASP-2 was the second protease component discovered after MASP-1 [6]. It shares the domain organization of MASP-1 and MASP-3 (Figure 2D). Among the serine protease components, it has the lowest abundance (about 0.5 μg/mL in serum). Still, it is an essential component of the LP, because it is the only component capable of cleaving C4 [44]. The second splice product of the *MASP2* gene is MAp19 (also known as MAP-2 or sMAP), a small two domain protein of 19 kDa. As for MAp44, an inhibitory role is attributed to MAp19.

### 2.3. The Sites of Synthesis

Components of the lectin pathway are mainly synthesized in the liver, but there are some exceptions [45]. In addition to the liver, pronounced expression of MBL-C has been detected in mouse intestinal epithelial cells [46]. Note that this form is absent in humans. H-ficolin is the most abundant PRM of the LP in humans, which is mainly synthesized in the liver, bile duct, and the lung. Interestingly, no equivalent is present in mice. L-ficolin is mainly produced by the liver, and M-ficolin is synthesized by immune cells, particularly monocytes [45]. Despite the name, CL-K1 is expressed not only in the kidney, but also in the liver and adrenal glands [23]. CL-L1 expression has been found in the liver, the adrenal glands and the placenta. CL-K1 and -L1 form heterocomplexes [12]; hence, a common site of their synthesis is presumable.

The main site of MASP-1 and MASP-2 synthesis is the liver. These two proteases are the drivers of the LP activation. MASP-3 has a distinct role and, not surprisingly, has a wide tissue distribution, including the liver, the colon, the bladder and the uterus [47]. The expression of MASP-3 is considered to be ubiquitous. Interestingly, the MASP-1 isoform is missing in birds and fish completely [48], while the ortholog of mammalian MASP-3 is present in many vertebrates. This might be due to the developmental role of MASP-3 (see later). MAp44 is mainly expressed in the heart, where it might have a protective role from LP-mediated tissue damage [10,41]. Finally, MAp19, just like the other splice variant of the *MASP2* gene, is mainly expressed in the liver [42].

## 3. Activation Mechanism of the Lectin Pathway

**Figure 3:**
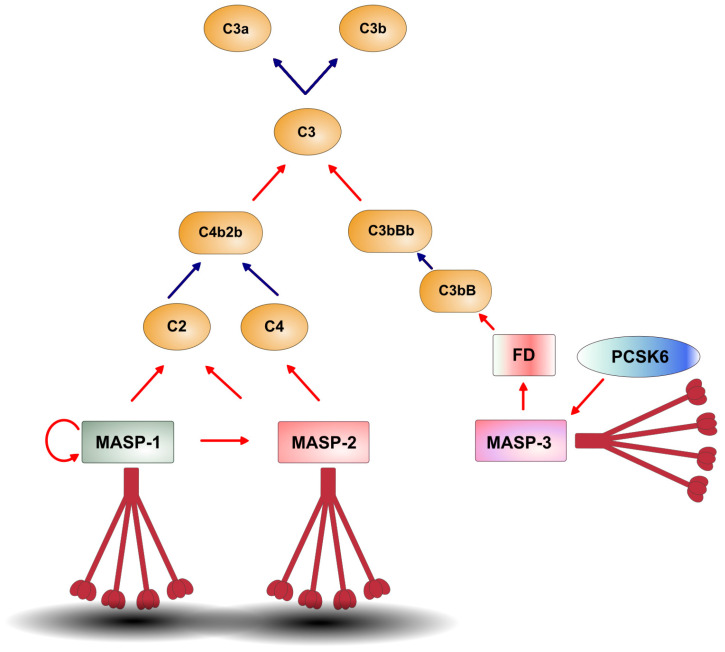
Activation mechanism of the LP and AP. The figure shows the activation mechanism of the LP and the initial activation steps of the AP. The two activation routes are closely connected. Clustering of PRM/MASP-1 and PRM/MASP-2 complexes on the activation surface triggers LP activation. AP activation is initiated in the fluid phase via the cleavage of MASP-3 by PCSK6, followed by the cleavage of pro-FD by MASP-3. Red arrows indicate proteolytic cleavages, while blue arrows indicate non-enzymatic events. Brown color indicates a PRM, while the gray oval shapes represent an activating surface. Detailed description can be found in the text.

Since there are three protease components associated with the PRMs, the role of each one in the activation mechanism of the lectin pathway has been a subject of intensive research in the past. The concentration of MASP-2 (6 nM) is the lowest among the lectin pathway proteases in the human serum [39], but it is the only MASP which can cleave C4 [44]. MASP-2 is an extremely efficient C4 cutter (k_cat_/K_m_ = 2.97 × 10^7^ M^−1^ s^−1^), and it is more efficient than the protease of the classical pathway with similar specificity, C1s [49]. The explanation for the efficient C4 cleavage is that MASP-2 has multiple binding sites for C4. Besides the substrate binding pocket of the serine protease domain, there are exosites on the complement control protein domains (CCP1-CCP2) which are closely connected to the SP domain [50]. MASP-2 can also cleave C2. Therefore, it can generate the classical/lectin pathway C3 convertase (C4bC2b, formerly known as C4bC2a). MASP-2 can also autoactivate, but its autoactivation capacity is very low compared to the other autoactivating enzymes of the complement system, such as C1r and MASP-1 [51]. For a long time, the prevailing theory was that MASP-2 is the autonomous activator of the lectin pathway. Only a supporting role was attributed to MASP-1, since it can also cleave C2 and, in this way, facilitate the formation of the convertase complex [52]. However, experiments with a MASP-1-specific inhibitor [53] and MASP-1-deficient human serum [54] have proven that MASP-1 plays a cardinal role in the lectin pathway activation. According to the current model of lectin pathway activation, following the binding of the PRMs to the activation surface, the first enzymatic step is the autoactivation of MASP-1 (Figure 3). Activated MASP-1, then, is the exclusive activator of MASP-2. Inhibiting MASP-1 prevents MASP-2 activation entirely and permanently. Although isolated MASP-2 in high concentration can autoactivate [55], this phenomenon does not manifest in human blood. The reason is that the physiological concentration of MASP-2 is very low, and because the serum concentration of MASP-1 (143 nM) is about 20 times higher than that of MASP-2, every MASP-2 molecule is surrounded by multiple MASP-1 molecules on the activation surface. Kinetic measurements have also corroborated the central role of MASP-1 in the lectin pathway activation [51]. The catalytic efficiency (k_cat_/K_m_) of zymogen MASP-1 autoactivation (4.5 × 10^2^ M^−1^ s^−1^) is three orders of magnitude higher than that of zymogen MASP-2 (0.14 M^−1^ s^−1^). In the second phase of the autoactivation, when active proteases cleave zymogen proteases, MASP-1 also outperforms MASP-2. Active MASP-2 cleaves its own zymogens with moderate efficiency (6.0 × 10^2^ M^−1^ s^−1^), while MASP-1 cleaves zymogen MASP-2 much more efficiently by two orders of magnitude (1.2 × 10^4^ M^−1^ s^−1^). The first evidence for the central role of MASP-1 in the lectin pathway activation was provided by experiments with sera of gene knockout (*MASP1/3*^−/−^) mice [56]. In these mice, both MASP-1 and MASP-3 were absent. Although the experimental data clearly showed no lectin pathway activity in the serum of these animals, the authors insisted that there is a low level of C4 deposition on mannan-coated microtiter plates. The addition of recombinant MASP-1 to these sera completely restored the lectin pathway activity. Furthermore, 10 years later, the same research group made gene knockout mice mono-specifically deficient in MASP-1 or MASP-3 [57]. They found that the MASP-1-deficient mice totally lacked lectin pathway activity, definitively confirming the central role of MASP-1 in this process.

Since MASP-3 cannot cleave any components of the lectin pathway, it has no role in the activation of this pathway. At the time of its discovery, an inhibitory role was attributed to MASP-3, as it binds to the same PRMs as MASP-1 and MASP-2 [8]. Since the serum concentration of MASP-3 is relatively high (63 nM), it really can slow down the activation process, similar to the two non-proteolytic components, MAp44 (41 nM) and MAp19 (22 nM). Moreover, since MASP-1, MASP-3 and MAp44 are the alternative splice products of the same *MASP1* gene, and these proteins contain the same interacting N-terminal region (CUB1-EGF-CUB2), they can form heterocomplexes, too [15]. The presence of these components on the activation complexes further reduces the chance that MASP-2 zymogen autoactivation can take place. On the other hand, since MASP-3 is an active serine protease, it has other important biological roles beyond the lectin pathway.

During lectin pathway activation, MASP-1 must contact MASP-2 on the activation surface. Theoretically, this can happen in two ways: either inside the PRM-MASPs complexes or between complexes. Although co-complexes with MASP-1 and MASP-2 may exist [58], only hexameric MBL (or ficolin) molecules are large enough to accommodate two protease dimers at the same time. Hexameric MBL can be detected in serum, but the predominant oligomeric forms are the trimers and tetramers [13]. It is more likely, therefore, that a PRM typically carries only a single MASP homodimer [59]. Intercomplex activation can take place on the activation surface where spatial arrangement (clustering) of the PRM-MASP-1 and PRM-MASP-2 complexes ensures the juxtaposition of MASP-1 and MASP-2 [60]. In this way, the activation of the lectin pathway is significantly different from that of the classical pathway, where the activation is thought to take place inside the C1 complex. Although there is a functional analogy between C1r and MASP-1 and also between C1s and MASP-2, there are important differences. C1r and C1s are present in equimolar amounts in the blood, while MASP-1 is much more abundant than MASP-2. C1r cleaves only C1s, while MASP-1 activates MASP-2 and C2 and has substrates outside the lectin pathway as well.

## 4. Connection to the Other Pathways of Complement

### 4.1. Connection to the Classical Pathway (CP)

There are obvious structural and functional similarities between the initiating complexes of the CP and the LP, and both pathways result in the formation of the C4bC2b convertase. Interestingly, complement activation by MBL was first demonstrated by adding the C1r_2_s_2_ tetramer to it, and MBL was thought to activate the classical pathway via this mechanism [4,61,62]. The possible in vitro interactions between human C1q, rat MBL-A, human MBL, rat ficolin-A, and MASP-1, MASP-2, C1r, C1s and their fragments were most extensively characterized by Phillips et al. [63]. The result was that the interactions of C1q with MASPs or that of MBL with C1r and C1s are possible with typical K_D_s of around 100 nM. The cognate MBL-MASP and C1q-C1r_2_s_2_ interactions are much stronger with K_D_s of about 2–4 nM. Now, it is established that PRMs of the LP preferably form complexes with MASPs and MAps [5,6,8,64], but the existence of cross complexes (i.e., C1q-MASP or LP PRM-C1r-C1s) cannot be ruled out completely in the blood. If cross complexes occur in the circulation, they probably constitute only a small fraction under normal circumstances in healthy individuals.

### 4.2. Connection to the Alternative Pathway (AP)

More intriguing is the connection of the LP to the AP. In the past decade, due to the work of several groups, it has become clear that MASP-3, a component of the LP, functionally belongs to the AP [57,65,66,67]. MASP-3 circulates predominantly as an active enzyme [68], and it cleaves and “activates” pro-factor D (pro-FD), producing mature factor D (FD) (Figure 3). Though it has been demonstrated recently that pro-FD has considerable enzymatic activity on its natural substrate, C3bB, this activity is 800-fold less than that of mature FD [69,70]. Therefore, mature FD is required for efficient AP activation, and so is the activated form of MASP-3. The activation mechanism of MASP-3 was also elusive until recently, when it was demonstrated that it occurs in the blood, and this fluid-phase activation is mediated by a secreted pro-protein convertase, particularly PCSK6 [71].

A role for MASP-1 has also been demonstrated in AP activation. It has been shown that a specific MASP-1 inhibitor, SGMI-1, could prevent the activation of the AP on certain activating surfaces, particularly LPS using the standard Mg^2+^-EGTA buffer system for AP measurement [72]. The mechanism for this phenomenon is still unknown. MASP-1 might be associated with ficolins, which can potentially bind to ligands in the absence of Ca^2+^. This could explain the surface attachments of MASP-1 in Mg^2+^-EGTA. C3 cleavage by MASP-1 might be the next step, but MASP-1 can only cleave the hydrolyzed form of C3, known as C3(H_2_O), efficiently [44,73]. Early reports demonstrated the involvement of MBL in AP activation in C2-deficient serum in a Ca^2+^-containing buffer [74,75]. It is possible that it was actually MASP-1 associated with MBL that caused this phenomenon. MASP-2 has been demonstrated to activate the complement system in C4- and C2-deficient serum [76,77,78]. This so-called “C4/C2 bypass” route obviously cannot proceed via the canonical activation path of the LP, which involves both C4 and C2. The mechanism of the C4/C2 bypass route is unknown, but it may proceed via the AP requiring direct C3 cleavage [78]. All in all, while the involvement of one LP component, MASP-3, in AP activation is well-established, further research is required for the elucidation of the contribution of other LP components.

## 5. Regulation of the Lectin Pathway by Natural Inhibitors

### 5.1. Inhibitors of Lectin Pathway Proteases

**Figure 4:**
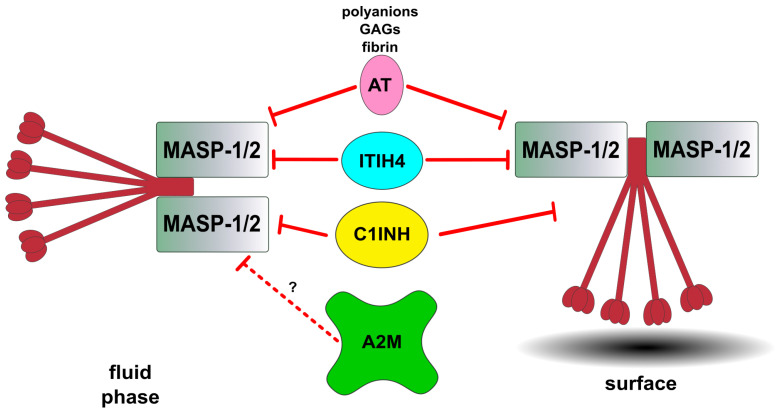
Regulation of the LP by natural inhibitors. The figure shows the physiological inhibitors of MASP-1 and -2. AT, ITIH4 and C1INH inhibit these proteases both in the fluid phase and activation surfaces, preventing (auto)activation. A2M may inhibit in the fluid phase. However, on the activation surface, it has no detectable effect on the LP. T-shaped lines refer for inhibition, while dashed T-shaped line refers to potential inhibition. Brown color indicates a PRM, while the gray oval shape represents an activating surface. Detailed description can be found in the text.

The complement system’s activation happens as a series of proteolytic events; therefore, it must be precisely and tightly regulated by natural inhibitors, which employ distinct strategies. Inhibitors may compete with substrates for the enzyme’s active sites. Furthermore, some utilize secondary binding sites, or even act as so-called “suicide substrates”, when they covalently bind to the protease active center, hence preventing any further catalysis. Among the plasma inhibitors, C1 inhibitor (C1INH), antithrombin (AT), alpha-2-macroglobulin (A2M), and inter-alpha-trypsin inhibitor heavy chain 4 (ITIH4) have been implicated in modulating the lectin pathway (LP) proteases (Figure 4) [79,80].

C1INH, encoded by the *SERPING1* gene, is a highly glycosylated acute-phase protein of around 71 kDa [81], but due to its heavy glycosylation, it runs at around 105 kDa on SDS-PAGE. C1INH plays a crucial role as a natural inhibitor of the lectin pathway by making covalent complexes with the target proteins after its reactive loop is processed by the target protease. The molecule can inhibit active MASP-1 and MASP-2, suggesting a significant regulatory function within the LP [79]. Beyond its function in the LP, C1INH also inhibits C1s and C1r, proteases of the classical pathway [82]. Moreover, C1INH has an impact on other biological pathways, including the modulation of the coagulation pathway, fibrinolysis and the contact system, underscoring its multi-faceted role in physiological regulation [83].

Antithrombin (AT) as C1INH is also a serpin which is encoded by *SERPINC1,* but in contrast to C1INH, it is a negative acute-phase protein, which means its level decreases during inflammation. It is a glycoprotein with a molecular mass of about 58 kDa [84]. As its name suggests, AT was first described as the inhibitor of thrombin, and other coagulation proteases, but as MASP-1 has a thrombin-like specificity [85], and both MASP-1 and MASP-2 were shown to convert prothrombin to thrombin [86,87], it is not too surprising that AT is also an inhibitor of MASP-1 and MASP-2 activities. In line with this, AT seems to be an equally potent inhibitor of the LP as C1INH, but only in the presence of heparin [79].

Alpha-2-Macroglobulin (A2M), encoded by the *A2M* gene, is a highly abundant plasma glycoprotein, which is also considered to be an acute-phase protein. A2M belongs to the C3/C4 protein family [88]. The protein consists of four identical subunits, each weighing around 180 kDa, totaling about 720 kDa. A2M’s inhibitory mechanism is often described using the “venus-flytrap” metaphor. Upon binding a protease active center via its bait region mimicking the protease substrates, the proteolytically processed molecule undergoes in a conformational change and fully encapsulates one or two proteases molecules, thereby impeding its activity toward natural macromolecular substrates. However, the protease retains its activity against small substrates [89]. This unique mechanism positions 2M as a pivotal regulator of proteolytic activity, which can inhibit MASP-1 and MASP-2, respectively, in vitro [44]. On the other hand, it seems that A2M is not a physiological inhibitor of the LP because no inhibitory effect was observed towards LP activation on mannan-coated surfaces [79].

Inter-alpha-trypsin inhibitor heavy chain H4 (ITIH4) is an acute-phase protein encoded by the *ITIH4* gene. As with the previous ones, ITIH4 is also glycosylated, and thus the molecule has a final molecular mass of around 120 kDa [90]. ITIH4 inhibits MASP-1 and MASP-2 by forming non-covalent complexes after proteolytic cleavage [80]. In these complexes, ITIH4 competitively prevents natural substrates from forming interactions with their proteases. However, small substrates can be processed after the complex formation, which highlights the fact that ITIH4 does not completely block the active center, but in contrast to A2M, it just covers a small surface on the MASPs, which enables the protein to be a solid-phase inhibitor of the LP. Despite its experimentally proven ability to inhibit the LP, uncertainties persist regarding its status as an in vivo inhibitor of the LP, as C1INH exhibits superior inhibitory capabilities.

Both C1INH and AT exert strong inhibitory effects on the lectin pathway, underscoring their potential as physiological inhibitors. The inhibitory potential of natural LP inhibitors is summarized in Figure 4.

### 5.2. Factor I, the Common Regulator of the Three Pathways

Factor I (FI) is a serine protease which degrades C3b and C4b in the presence of cofactors. Consequently, it is a common inhibitor of all three complement pathways at the level of C3 convertases [91,92]. FI circulates as a cleaved, pre-activated protease (like MASP-3 and FD) in human blood. The cleavage of FI and MASP-3 is mediated by proprotein convertases. However, FI is supposed to be cleaved during the secretion, while zymogen MASP-3 is converted in the circulation [93]. Although FI is cleaved (an RRKR tetrapeptide is missing), it can exert its proteolytic activity only in the FI/cofactor/substrate ternary complex [94]. Factor H is a cofactor for only C3b degradation [95], while the C4b-binding protein (C4BP) is the main cofactor for C4b degradation [96]. Other cofactors such as the membrane cofactor protein (MCP or CD46) and complement receptor 1 (CR1 or CD34) are involved in both C3b and C4b degradation. Similar to MASP-3 and FD, FI has no known physiological inhibitor. The enzymatic activity of these proteases is regulated though their extremely narrow and cofactor-dependent substrate specificity. Another membrane protein, decay-accelerating factor (DAF or CD55), is not a cofactor for FI, but it is also a common inhibitor of the complement activation pathways by preventing the formation and accelerating the decay of the C3bBb and C4bC2b convertase complexes. A deficiency or mutation of FI is associated with various disease conditions such as bacterial (both gram-positive and gram-negative) infections, glomerulonephritis, age-related macular degeneration and atypical hemolytic uremic syndrome [97,98,99].

## 6. Direct Cellular Effects of Lectin Pathway Components

As we will describe later, the components of the LP have frequently been studied in various diseases. In contrast to this, there are relatively little data on the in vitro cellular effects of these proteins. Moreover, pattern recognition molecules (PRMs) of the LP have been more intensively studied in this respect than the associated proteases.

### 6.1. Cellular Effects of PRMs

MBL has at least two well-defined cell surface receptors. One of them is CD93 (C1qRP), which is a common receptor for C1q and SPA besides MBL [100]. The other one, calreticulin (cC1qR), is able to bind several proteins with a collagen-like domain (e.g., C1q, CL-K1) and induces signaling in different cell types [101,102] upon MBL (or CL-K1) binding. The effects of MBL on different cell types are greatly diverse, which may suggest the utilization of other receptors besides CD93 and calreticulin. Anti-inflammatory characteristics of MBL have been found in mammals [101,103,104] as well as in fish [105], suggesting that the interaction of soluble PRMs with cellular components of the immune system is an evolutionary conserved mechanism. Anti-inflammatory properties of MBL include the suppression of pro-inflammatory cytokines and the inhibition of T cell proliferation through calreticulin. On the other hand, pro-inflammatory effects of MBL have also been shown, as it increases the IL-1β, IL-6 and IFNγ production of mature human dendritic cells (DCs) [106] and enhances the plasmacytoid-to-DC differentiation and their pro-inflammatory cytokine production [107]. MBL also induces pro-inflammatory responses in platelets [108] and in corneal epithelial cells [109], where the involvement of IL-1 signaling is a common factor in both studies. Various cellular effects of MBL that do not involve inflammation directly have also been described. It induces cytotoxicity in human tubular epithelial cells [110] and senescence in hepatic stellate cells [102] and inhibits osteoclast differentiation from monocytes [111]. Although endothelial cells can bind MBL through the C1qRP, we could not find any downstream effects of MBL on these cells [112].

Whereas both the pro-inflammatory and anti-inflammatory cellular effects of MBL have been demonstrated, the situation seems much more straightforward in the case of ficolins and collectins. Ficolin-2 induces macrophage polarization into the M1 direction and DCs to present antigens to CD8^+^T cells more effectively [113,114,115]. In contrast, CL-K1 promotes M2 macrophages differentiation and directly enhances melanoma proliferation in a murine model [116].

### 6.2. Cellular Effects of MASPs

There are less data on the cellular effects of the PRM-associated serine proteases of the LP. MASP-1, the most abundant enzyme of the LP, directly activates endothelial cells by cleaving protease-activated receptors (PARs, PAR1, 2 and 4), initiating the Ca^2+^, NF-κB, p38 MAPK, CREB and JNK signalling pathways in HUVECs [117,118]. Upon the activation of these pathways, endothelial cells undergo several morphological and functional changes, resulting in a characteristic proinflammatory phenotype, defined by proinflammatory cytokine production, altered adhesion molecule pattern and increased permeability. In HUVECs, MASP-1 induces the secretion of IL-8, predominantly through the p38-MAPK pathway, and increases E-selectin expression, facilitating neutrophil chemotaxis and enhanced adhesion to the endothelium [118,119]. Moreover, MASP-1 has been found to directly stimulate neutrophils through neutrophil surface receptors, leading to an increased oxidative burst and enhanced phagocytosis in goat [120]. The cleavage of zymogen MASP-1 into the active form is a prerequisite for its direct cell-activating effect [121]. Given that MASP-1 activation can be triggered by bacterial infection or tissue damage, its capacity to promote neutrophil activation and endothelial cell-driven neutrophil recruitment to the site of inflammation or injury serves as a highly effective defense mechanism. MASP-1 induces endothelial permeability via the paracellular route in HUVECs utilizing PAR1-mediated intracellular Ca^2+^-mobilization, Rho-kinase activation-dependent MLC phosphorylation, cytoskeletal actin rearrangement, and disruption of interendothelial junctions [122]. On the one hand, increased permeability during inflammation can facilitate the extravasation of soluble immune mediators and the transmigration of immune cells to the underlying tissue, contributing to the elimination of infectious agents and tissue debris. On the other hand, however, pathologically increased vascular permeability underlies several potentially life-threatening diseases, such as hereditary angioedema, sepsis and ischemia/reperfusion [123]. Further underscoring the relevance of MASP-1 in inflammation, it was found to strongly synergize with other commonly recognized proinflammatory mediators (LPS, histamine, IFNγ, and bradykinin) [124] and with hypoxia (an unpublished observation) to enhance the inflammatory response of HUVECS. The proinflammatory cell-activating effect of MASP-1 extends to other cell types besides endothelial cells and neutrophils. MASP-1 promotes the transition of quiescent hepatic stellate cells into an activated state through p38-MAPK/ATF2 signaling, thereby promoting liver fibrogenesis [125,126]. It has been shown that in fish (*Nile tilapia*), a *MASP1* gene product, the ortholog of human MASP-3, induces early expression of IL-6 and IL-8 in macrophages, enhancing the inflammatory response, whereas later it increases their IL-10 and MIF expression, mitigating inflammation and restricting the migratory capacity of phagocytes [127]. As fish lack MASP-1, the effects of LP proteases in fish cannot be automatically translated to the human system. Besides its inflammation-related effects, MASP-1 activates platelets in a PAR4- and thrombin-dependent manner, possibly contributing to the physiological coagulation response [128], which will be detailed in the next section.

The direct cellular effects of MASP-2, another key serine protease of the LP besides MASP-1, is even less characterized. We showed that MASP-2 induces Ca^2+^ signaling, disrupts intercellular adhesion junctions and increases endothelial permeability in HUVECS similar to MASP-1, implying a PAR-mediated direct endothelial cell-activating effect [129]. Furthermore, the inhibition of MASP-2 by narsoplimab, an anti-MASP-2 human monoclonal antibody, reduces microvascular endothelial cell injury by decreasing caspase 8 activation, downregulating the gene expression of various proinflammatory and proapoptotic proteins, and concurrently upregulating the expression of anti-inflammatory and pro-angiogenic factors [130,131].

**Figure 5:**
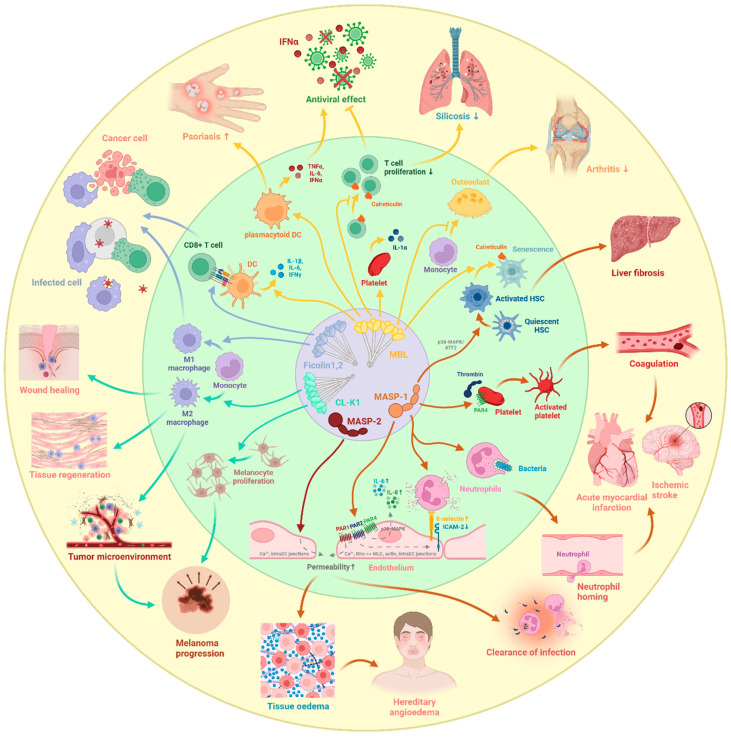
Direct cellular effects of LP components. In the central blue circle, only those key LP components, including pattern recognition molecules (MBL, Ficolin-1, -2, CL-K1) and serine proteases (MASP-1, MASP-2), are highlighted which were demonstrated to have direct cellular effects. Surrounding this core, the green circle outlines the immediate outcomes of these interactions with cells. The outer yellow circle illustrates the broader physiological/pathophysiological consequences resulting from the direct cellular effects of LP components. Direct molecular interactions between LP components and cells are indicated by plain arrows, emphasizing the initial impact. Subsequent, non-direct consequences are represented by faded lines, highlighting the intricate cascade of events triggered by these interactions. This figure provides a comprehensive visualization of the complex network and outcomes associated with LP components, shedding light on their diverse cellular effects. The color of the different arrows is the same as the color of the LP component initiating the effect. Created with BioRender.com.

Collectively, the direct cell-activating properties of LP serine proteases are evidenced across different cell types, mainly pointing to a proinflammatory direction (Figure 5). It connects the antimicrobial effects of the quickest-responding complement system and that of the highly effective neutrophil granulocytes via endothelial cells, and it reprograms hepatic function.

## 7. Influence of Lectin Pathway Components on Coagulation

The complement system and the coagulation–fibrinolytic cascades are the two main proteolytic networks in the blood of vertebrates. For practical reasons, they treated as separate systems. However, when activated, proteases of one system can also cleave components of the other. Activation of the two cascades often occurs simultaneously and potentially reinforce the impact of the other. Interestingly, C3, the most abundant complement protein, was found to be present in blood clots, and a higher C3 content correlated with prolonged fibrinolysis time [132]. Another recent study also supports the involvement of complement in coagulation in multiple ways [133]. An excellent review dealing with all aspects of complement and coagulation interconnectivity has been published recently [1]. In this section, we will concentrate on published results regarding the involvement of LP components in coagulation and fibrinolysis.

Most such papers focus on two proteases components, MASP-1 and MASP-2. Both MASP-1 and MASP-2 have been shown to cleave coagulation factors in vitro. Initially, MASP-2 was shown to activate prothrombin but with a low efficiency [87]. Later, MASP-1 was shown to cleave fibrinogen, FXIII, prothrombin, and TAFI (thrombin-activatable fibrinolysis inhibitor, also known as carboxypeptidase B2) [134,135]. These cleavages have also low efficiencies compared to those of the cognate activators, FXa and thrombin. While the cleavage patterns are also somewhat different, active fragments are produced by MASP-1, and MASP-2. Nevertheless, in turbidimetric and thromboelastographic assays using human plasma or whole blood, added recombinant MASP-1 has shown a procoagulant effect by reducing the clotting time, and this effect was dependent on the presence of prothrombin [86,135,136]. Interestingly, the presence of MASP-1 has proven to have an influence on the structure of the fibrin filaments shown on electron microscopic images [135]. This might be due to the direct activity of MASP-1 on fibrinogen or FXIII.

A close-to-physiological setup to study coagulation in human blood is a microvascular whole blood flow model using “endothelialized” microcapillaries [137]. Another useful tool is to use specific inhibitors, which allows one to assess the effects of endogenous MASP-1 and -2 at a physiological concentration. SGMI-1 and SGMI-2 are two such inhibitors for MASP-1, and MASP-2, respectively [53]. Of the two inhibitors, only SGMI-1 had a significant effect on coagulation, as it reduced the rate of fibrin clot formation in the microvascular whole blood flow model, while SGMI-2 had no significant impact [128,138]. Previously, SGMI-2 was also ineffective in thrombelastometric measurements [136]. The involvement of MBL was also demonstrated in the microvascular whole blood flow model system. MBL was shown to co-localize with von Willebrand factor, fibrin and activated platelets, whereas MASP-1, together with thrombin, might further activate platelets by cleaving PAR4 [128]. The impact of MASP-1 on fibrinolysis was also examined in the same model and with purified components. MASP-1 cleaved tPA and plasminogen, although with a low efficiency. In the microvascular whole blood flow model, significant inter-individual differences were observed [139], delineating the complexity of the impact of MASP-1 on fibrinolysis.

It has been shown that platelet activation and fibrin formation results in increased levels of MASP-1/C1INH and MASP-2/C1INH complexes and also complexes with antithrombin (AT). In this study, ficolins, rather than MBL, were shown to bind to activated platelets. The increased levels of MASP–serpin complexes were associated with thrombotic markers in systemic lupus erythematosus (SLE) patients with thrombotic disease [140]. These results might indicate that a positive feedback could exist in coagulation and LP activation.

The impact of LP components was also demonstrated in vivo, using mouse models for coagulation and certain coagulopathies. MBL-null knockout (KO) mice, as well as MASP-1/3-null KO mice both demonstrated prolonged bleeding time in a tail clip bleeding experiment [141]. On the other hand, MBL-null mice developed disseminated intravascular coagulation (DIC) when infected with Staphylococcus aureus, showing that MBL is protective in this vascular complication. In the same models, MBL-null mice, as well as MASP-1/3-null mice had reduced thrombogenesis in the FeCl_3_-induced model [142]. Using a different approach, MAp44 (also known as MAP-1), an inhibitor of the LP by displacing MASPs from PRMs, was shown to have a protective role against thrombogenesis in the FeCl_3_-induced model and resulted in reduced infarct size in the coronary-artery-ligation-induced myocardial infarction model in mice [143]. Finally, patients with low MBL levels had a reduced risk for future venous thromboembolism including deep vein thrombosis, showing that the involvement of the LP in coagulation could be indeed clinically relevant. The impact of MBL in this scenario was thought to be mediated by MASP-1 [144].

To summarize these studies, MASP-1, rather than MASP-2, seems to be more important in fibrin clot formation. Direct MASP-1 effects might have an influence on the structure of the fibrin clot, rather than the clotting time. In this respect, the indirect effect of MASP-1 via prothrombin activation could be more important, leading to faster clotting. The effect of MASP-1 is probably mediated by MBL targeting MASP-1 to the coagulation site, possibly via the von Willebrand factor. Ficolins might also play a role in targeting MASPs to activated platelets, but further research is required to uncover the mechanistic details of the interplay between the LP and coagulation.

## 8. Diseases with Potential Lectin Pathway Involvement

Detrimental activation of the complement system is known to underlie many diseases. In most cases, more than one pathway is activated at the same time, and it is difficult to distinguish which plays a greater role in pathological processes. However, there are diseases in which the activation of the lectin pathway is of particular importance (Table 1). We will go through these in the following subsections.

### 8.1. Renal Diseases

Probably the most affected part of human body by pathological complement activation is the kidney. Since kidney cells can produce complement components, the entire repertoire of the complement cascade is available locally, while blood circulation can also provide active complement elements. Unwanted activation of the lectin pathway is often not the primary driver of glomerular diseases but a consequence of the presence of injured kidney cells [197].

#### 8.1.1. IgA Nephropathy

*IgA nephropathy* (*IgAN*) is a severe primary glomerulonephritis, which is a leading cause of renal failure. It is now treated as an autoimmune disease that is initiated by flawed IgA1 synthesis and abnormal glycosylation of the heavy chain. Galactose-deficient IgA1 accumulates in the circulation that leads to a complex formation with autoantibodies. Deposits develop in the glomeruli causing mesangial cell proliferation [198]. The most characteristic symptoms of IgAN are hematuria, proteinuria, and acute kidney injury. These processes eventually lead to chronic kidney diseases. The pivotal role of complement in the progression of the IgAN is well known, and glomerular co-deposition of IgA1 and complement components, mostly C3, have been observed in biopsy samples. Lectin pathway involvement has been proven via the detection of C4d deposition in renal biopsies without C1q present [145]. The initiation of the LP is triggered by pattern recognition molecules binding to the aberrantly glycosylated IgA1. Newly exposed N-acetylgalactoseamine serves as target to MBL and ficolin-2. Another explanation for LP activation can be the binding of MBL to neoepitopes on the surface of injured glomerular cells. MBL and MASP-1 have been detected in 24% of IgAN patients along with C3b/C3c and C5b9, but a correlation with the severity of IgAN could not be established [146]. Roos et al. also found MBL, ficolin-2, MASP-1, MASP-3 and C4d deposits in 25% of the patient group. These patients suffered more serious renal damage such as increased mesangial proliferation, glomerular sclerosis, interstitial infiltration, and proteinuria [147]. Data from a large Spanish cohort presented C4d deposits in 38.5% of the patients that correlated with the development of end-stage kidney disease [199]. Variations in LP components in the circulation were measured, but a correlation with IgAN severity was not consistent among different groups. Guo et al. [148] presented data that showed that low MBL levels indicated more serious IgAN, while Roos et al. [147] could not verify the connection. Plasma concentration of ficolins-1, -2, MBL, MASP-1 and MAP-19 significantly increased, while the level of MASP-3 decreased in IgAN patients. A low level of MASP-3 accompanied by an increased level of C3 cleavage products and FHR5 indicated progressive disease [149]. Clinical observations suggest that IgAN is mostly triggered by LP activation, while aggravation of the disease might be due to the participation of the AP.

#### 8.1.2. Membranous Nephropathy

Aberrant glycosylation of IgG4 gives rise to the activation of the lectin pathway in another rare but serious glomerulonephritis, the *membranous nephropathy* (*MN*). The major activators in primary MN have been proven to be antibodies that bind to the phospholipase A2 receptor (PLAR2) on podocytes [157]. These IgG4 type antibodies do not activate the CP, but the co-localization of MBL, C3 and C4d on the subepithelial surface of glomerular capillaries indicates activation of the LP [153]. PLAR2-seropositive patients present elevated levels of MBL and C4d in serum and in urine, which seems to correlate with proteinuria [154]. It has been shown that patients with anti-PLAR2 antibodies are more prone to reach remission if they had initially lower serum levels of MBL, MASP-1 and MASP-2 [155]. Another MN-causing antigen is the thrombospondin type 1 domain-containing 7A (THSD7A). The LP-activating mechanism is similar to that of PLAR2. Human anti-THSD7A antibodies are able to raise the levels of MBL, MASP-1 and -2 in mice [156].

#### 8.1.3. Diabetic Kidney Disease

One of the most common causes behind end-stage renal failure worldwide is the slow inflammation in the kidney caused by diabetes mellitus type 1 or 2. There is an enormous amount of data in the literature dealing with the role of the LP in diabetic kidney disease (DKD) [200]. Unwanted initiation of complement activation is due to the enzymatic or non-enzymatic glycosylation of proteins, lipids, and nucleic acids in the hyperglycemic environment. This phenomenon can affect the complement cascade in two ways. The first is the activation of the LP through MBL and ficolin-3 binding to these new neo-epitopes, while the other is the uncontrolled complement activity due to hyperglycosylated regulatory proteins losing their function. The data showed that levels of MBL, C4d, C3a and four other complement components significantly increased in the glomeruli, arterioles and urine of DKD patients compared to diabetic patients who did not suffer from renal damage [158,159].

### 8.2. Ischemia/Reperfusion Injury

#### 8.2.1. Renal Ischemia/Reperfusion Injury

The vital role of complement is now generally accepted in *ischemia reperfusion injury (IRI)* in several organs such as the kidney, heart, intestine, liver, skeletal muscle, and brain. Ischemia develops when blood flow is permanently obstructed in a certain tissue that is followed by reperfusion when blood flow returns to the affected area. Ischemia and reperfusion initiate inflammation-like processes that cause severe tissue damage. Complement activation occurs at the early stage of IRI locally, and the activated complement components such as C3a, C5a and sC5b-9 appear in the circulation ahead of clinical symptoms [201]. The recruitment and activation of neutrophils by anaphylatoxins and the effect of MAC lead to the complement-mediated tissue injury that can spread to tissues initially not ischemic. Lectin pathway components, especially the pattern recognitions molecules collectin-11 (CL-K1), MBL and their enzymatic partner, MASP-2, seem to play key roles in commencing undesirable inflammation after renal and cardiac reperfusion [161]. In kidneys, LP activation is suggested to be triggered by CL-K1, which is expressed at elevated levels by tubule cells after IR stress. MASP-2 and C3d were found to be colocalized with CL-K1 and its preferred ligand L-fucose on the surface of renal epithelial cells [162]. An alternative activation of the LP is suggested by Yaseen et al. [78], the so-called C4-bypass where MASP-2 initiates the LP without the cleavage of the C4 protein [76,77,78]. The researchers found this theory suitable for the LP activation in kidneys during IRI based on an animal model. MBL deficiency proved to be protective in a renal mouse model [163], but C4 knockout mice were not protected from IRI [164]. Nevertheless, data are not available in human patients to further extend this hypothesis. Complement activation during kidney transplantation is an intensively examined area, since transplanted organs experience IRI during implantation. Clinical data have shown that recipients who possessed lower serum levels of MBL were prone to avoid organ rejection [165].

#### 8.2.2. Myocardial Ischemia/Reperfusion Injury

Similar processes are initiated by the LP in the heart after myocardial infarction (MI). A shortage of blood supply generates damage-associated molecular patterns (DAMPs) and causes the damage of complement regulatory proteins on the endothelial cells, leading to the devastation of cardiomyocytes. LP activation plays an important part in the reperfusion phase of myocardial ischemia/reperfusion injury that is shown to increase the extent of infarct size by up to 50% [202]. The deposition of MBL and C3 in a rat heart was the first observation concerned with LP involvement [170], which was further demonstrated in other experimental models. LP activation undoubtedly plays a part in MI, but the limited number of patients makes human data less conclusive. According to Zhang et al., the level of MASP-2 in patients with open heart surgery dropped as low as 50% compared to those patients who had stable coronary artery disease (CAD) [171]. In another study, a lower concentration of MASP-2 and a higher concentration of MASP-1 were observed in myocardial infarction patients compared to the ones in CAD patients [172]. MASP-2 and ficolin-2 consumptions have been shown in several papers [173,174], but a correlation between the infarct size and the examined complement components could not be confirmed. The role of MBL is controversial. Bonnemeier et al. presented data that showed that 30-day mortality among MBL-deficient MI patients was significantly lower [175]. On the other hand, Holt et al. demonstrated that low levels of pattern recognition LP molecules provide no protection from the consequences of IRI. Furthermore, MBL and ficolin levels had no prognostic value concerning the short-term outcome of myocardial infarction [176]. MASP-1 might have an interesting part in the initiation of MI due to its clot generating ability [138]. Generally, more in vitro and in vivo data are needed to determine whether the activation of the LP is a cause or a consequence of myocardial infarction in humans.

#### 8.2.3. Ischemic Stroke

Stroke is the third leading cause of death in developed countries. A sudden change in blood supply initiates hypoxia and ischemic injury in a part of the brain, leading to tissue damage and acute neurological disfunctions. Changes in the levels of complement components in patients after stroke indicate the initiation of the complement cascade [203]. MBL-null mice were used in focal cerebral IRI to examine the contribution of the LP to the severity of stroke. A smaller infarction size, better functional outcome, significantly decreased C3 deposition, and neutrophil infiltration were measured, while reconstitution with human recombinant MBL enhanced brain damage [179]. Furthermore, in the case of stroke patients, the outcome was associated with the MBL genotype. Low MBL levels also meant lower levels of C3, C4, CRP and proinflammatory cytokine profiles and better recovery after 3 months [179]. The significant role of MBL in ischemic stroke was further reinforced by data showing a smaller infarct size and a more favorable outcome in MBL-deficient patients who received conservative treatment [180]. LP involvement was shown in a MASP-2 knockout mouse model of focal cerebral ischemia. Mice lacking MASP-2 developed reduced neurological deficits and infarct volumes compared to wild-type mice [181]. Tsakanova et al. found a correlation between the increased activity of MASP-1/-2 and the risk of ischemic stroke [182]. In the patients’ blood, a more pronounced cleavage of C2 and C4 was detected compared to that detected in the healthy controls. They postulated that some genotypes had a protective role against the development of stroke and post-ischemic brain damage.

### 8.3. Atherosclerosis

More evidence supports the idea that lectin pathway activity is related to atherosclerosis and atherosclerosis-related acute myocardial infarction (AMI). Chronic low-level inflammation in the vessels contributes to the generation, progression and also the destabilization of plaques. The presence of MBL and ficolins in a plaque environment indicates LP involvement. Vengen et al. showed that MBL deficiency correlated with the severity of atherosclerosis [183]. More recently, ficolin-1, 2 and -3 were found in carotid plaques, while MBL was present mostly in ulcerated plaques, where MBL-driven LP activation was shown to be associated with plaque instability [184]. Furthermore, a higher concentration of ficolin-2 and pentraxin-3 and a low level of MASP-3 might predispose individuals to develop AMI [185].

### 8.4. COVID-19

The complement cascade usually plays a major role in diseases that cause extensive inflammation, as is what happens in COVID-19 [204]. Shortly after entering the host, structural proteins of severe acute respiratory syndrome coronavirus-2 (SARS-CoV-2) appear in the circulation and in all body fluids, initiating an immense immune response. All three complement pathways proved to be active after the onset of COVID-19, and more severe symptoms correlated with more intense complement activation. Several findings have validated that the lectin pathway contributes in causing or aggravating symptoms, since activated LP components have appeared in plasma and biopsy samples of COVID patients. Increased plasma levels of the MASP-1/C1INH complex and C4d were shown in a large cohort, which indicated LP activation [186]. Furthermore, elevated MASP-2 concentration was measured by a specific ELISA in hospitalized patients that correlated with the levels of ficolin-2, -3 and the C-reactive protein [187]. The importance of MASP-2 was further demonstrated by its significant deposition in kidney and lung biopsy samples of deceased patients [188]. MBL is a central player in the interaction between the LP and viral proteins. Highly glycosylated spike (S) proteins can be recognized by MBL. Therefore, MBL-driven complement activity hinders virus entry at the early stage of infection [189]. On the other hand, MBL polymorphism does not influence the outcome of disease progression [186]. The most abundant viral protein in plasma is the nucleocapsid (N) protein which is thought to interfere with the LP. Gao et al. presented data that showed that the N-protein of SARS-CoV-2 could initiate the LP through binding directly to and activating zymogen MASP-2 [190]. They also showed that the N-protein was capable of enhancing the autoactivation of MASP-2. In vitro binding of MASP-2 and MBL to the N-protein was also demonstrated by Ali et al. [191]. Notwithstanding, these results are in contrast with the findings of Stravalaci et al. who could not confirm MBL and N-protein interaction [189]. According to the controversial experimental data and less conclusive clinical observations, the role of the LP in COVID-19 is still not fully understood.

### 8.5. Hereditary Angioedema (HAE)

Insufficient lectin pathway regulation by C1INH may contribute to the development of a rare but severe disease, hereditary angioedema (HAE). In HAE, mainly plasma kallikrein is responsible for the cleavage of high molecular weight kininogen and the release of the proinflammatory peptide, bradykinin. Kallikrein activity is regulated by C1INH, but mutations of the *SERPING1* gene lead to low plasma C1INH activity that impairs the control of both the contact activation and the complement system. The accumulation of bradykinin results in increased vascular permeability and intense tissue swelling. The cleavage of high molecular weight kininogen by MASP-1 and the release of bradykinin were shown by Dobó et al. in human plasma [193]. This serves as an example of the connections between the kallikrein–kinin and the lectin cascades. Clinical data showed that levels of MASP-1 and MASP-1/C1INH complexes were reduced in HAE patients, which correlated with the severity of the disease. Significant consumptions of MASP-1 and C4d were considered to be caused by LP activation [194]. Another contributing factor of MASP-1 to HAE could be the increased permeability of endothelial cells due to the action of this protease on PARs, as discussed in the “Cellular effects of MASPs” section.

### 8.6. Autoimmune Diseases: Systemic Lupus Erythematosus and Rheumatoid Arthritis

Impaired control of the complement system may lead to autoimmune diseases [205]. Systemic lupus erythematosus (SLE) and rheumatoid arthritis (RA) are among them, and increasing data support the participation of the lectin pathway in the onset of their symptoms.

SLE is a chronic, life-threatening, and complex immune-mediated disorder. The deposition of activated complement products in the tissue and hypocomplementemia indicate massive complement involvement, mostly through the activation of the CP by autoantigens. The role of LP components has recently begun to be investigated, and the mechanism has not been fully revealed. The serum levels of LP proteins differ from those of healthy controls. Ficolin-2, ficolin-3, MASP-2, and MASP-3 concentrations were higher in a large cohort of SLE patients [206]. The level of MBL seemed to be significantly elevated and correlated with the severity of the disease Furthermore, the results indicated MBL participation in the development of lupus nephritis in SLE patients [207]. Another study showed reduced levels of MASP-1 and MASP-2 in proliferative lupus nephritis and severe SLE without lupus nephritis, while in patients with membranous nephritis, the concentration of these serine proteases was the same as in healthy controls [208]. Functional ELISA assays further reinforced the activation of the LP, measuring independently the activation of the three pathways in SLE serum samples [209]. Serum levels of MASP-1/C1INH and C1s/C1INH complexes indicated that both the CP and LP are active in SLE. However, LP contribution is more substantial in lupus nephritis [210].

Rheumatoid arthritis is characterized by chronic inflammation of the joints. Anomalous complement function due to faulty control contributes to an autoimmune disease that attacks cartilage, synovium and finally bones [211]. Synovial fluid that represents local inflammatory situations contains activated complement factors in elevated concentrations [212]. The accumulation of the IgG glycoform that lacks galactose initiates not only the CP, but the LP through MBL binding to newly exposed N-acetyl glucosamine in the Fc region [213]. Ammitzboll et al. measured a higher level of MBL, ficolin-1, ficolin-3, MASP-2 and MASP-3 in the plasma and synovial fluid of RA patients. Furthermore, ficolin-1 concentrations correlated with the disease activity [214,215]. A collagen antibody-induced mouse RA model demonstrated an interesting new role of liver-derived MASP-3, namely, its contribution to the damage of joints affected by RA [216].

### 8.7. Schizophrenia

Schizophrenia is a severe mental disorder, which causes a range of various psychological symptoms. Symptoms can include hallucinations, delusions and disorganized thinking. The etiology of schizophrenia is unknown, but it seems likely that a combination of genetic and environmental factors contributes to the development of the disease [217]. It has also been suggested that immunological processes [218], and particularly the inappropriate complement activation, may also play a role in the etiopathogenesis [219]. Recently, a number of observations have suggested that the complement system plays important roles in the brain’s development [220]. The components of the lectin pathway are expressed in the brain, and they are involved in the neuronal migration during brain development [221]. Malfunctioning of the lectin pathway may contribute to the development of schizophrenia. Mayilyan et al. showed that the functional activity of the MBL-MASP-2 complexes is higher in the sera of schizophrenic patients compared with healthy individuals [59]. Recently, the same authors also showed that a significant increase in ficolin-2-bound MASP-2 activity was observed in schizophrenia [196]. Although it is difficult to estimate the exact contribution of the disturbed lectin pathway to the pathogenesis of such a complex disease, these results clearly indicate that an intact complement system is needed for healthy neurological development and functions.

### 8.8. Influence of Aging

Since the lectin pathway fights against the invading pathogens before the development of the adaptive immune response, it is not surprising that an intact lectin pathway is more important in early childhood than in adulthood. Deficiency of the lectin pathway components can result in recurrent infections in children [222,223,224]. The age variations of the concentration of lectin pathway components are in agreement with the above phenomenon. In the case of MBL and ficolins, the highest serum concentrations were detected in children (typically 1–16 years), while in neonates and in adults, these concentrations were significantly lower [225]. Among adults, only insignificant changes of lectin pathway protein concentrations were observed with age. This indicates that aging does not influence lectin pathway activity in adulthood [39].

## 9. Therapeutic Inhibition and Testing of the Lectin Pathway

### 9.1. Therapeutic Inhibition of the Lectin Pathway

Complement-related diseases can rarely be attributed to a single pathway. In most cases, the activation of all three pathways occurs at the same time and at the same place. Therefore, in general, the anticipated therapeutic requirement is the simultaneous inhibition of the entire system. Inhibitors against junction point components such as C3 (compstatin analogs) or C5 (anti-C5 monoclonal antibodies: eculizumab and ravulizumab) can satisfy these demands in many cases. However, total elimination of complement effector functions could be rather disadvantageous regarding side-effects such as increased susceptibility to infections, especially to those caused by Neisseria. On the other hand, long-term complement inhibition by eculizumab proved that serious infection occurred only in 5.9% of treated patients compared to 4.1% of the placebo group [226]. Concerning LP-related disorders, selective targeting of undesirable activation can be challenging. Functional redundancies, high structural and enzymatic activity profile similarities make rational drug design a rather complicated task. Pathway-specific blockages of initiation can be carried out at the level of early LP components, i.e., the pattern recognition molecules (MBL, ficolins) or the serine proteases (MASPs).

The natural inhibitor of the LP, C1INH, can be adopted as a therapeutic agent. Although not selective for MASP-1 and -2, since it also blocks the classical pathway and proteases of the contact system, it is already used successfully in HAE treatment, while there are ongoing preclinical trials for myocardial and renal IRI. HAE patients have been treated with C1INH as a replacement therapy for decades [227]. Improved recombinant and isolated forms are available from various companies (see Table 1). C1INH treatment extenuates the intensity of acute attacks, and in prophylaxis, it significantly reduces the frequency of severe events [228]. The main target protease in the case of HAE is the plasma kallikrein, but C1INH can also prevent the MASP-1-mediated bradykinin release as well [193]. Promising results were achieved in various animal models of renal IRI; C1INH pretreated mice and non-human primates showed better survival rates and more rapid renal function recovery [166,167]. C1INH application in organ preservation solutions was able to reduce inflammation in transplanted organs [229]. The administration of C1INH for patients in renal transplantation improved kidney functions and reduced graft rejection in short- and long-term studies [230,231]. Since there are no approved complement inhibitors for the treatment of IRI, C1INH could have a new indication to attenuate the LP in acute myocardial infarction. Being a participant of other proinflammatory processes, C1INH still holds an untapped potential to become a beneficial treatment for IRI. It might be used in combination with new drugs of different mechanism to reduce infarct size and to mitigate tissue damage [202].

MASP-2 as a key activator of the LP seems to be a very advantageous target regarding specific LP inhibition. The relatively narrow substrate specificity of MASP-2 provides an opportunity to block the LP exclusively. Furthermore, in contrast to MASP-1, MASP-2 has a low serum concentration, is not involved in the contact activation and has a moderate effect on endothelial cells. Therefore, future therapeutic side-effects could be minimized. MASP-2 inhibitors are at different stages of development. One monoclonal antibody is already at clinical trials, while recombinant small protein inhibitors are tested in ex vivo models.

Narsoplimab, a fully human monoclonal anti-MASP-2 antibody produced by Omeros (Seattle, WA, USA), has been the most advanced-stage candidate for treating LP-related diseases so far. It expressed cardioprotective potential in a myocardial infarction mouse model by significantly reducing infarct size [150]. Narsoplimab was indicated in membranous nephropathy and reached phase II clinical trial [157]. A very promising therapeutic direction was the treatment of IgA nephropathy, but phase III clinical trials (ARTEMIS-IGAN, NCT03608033) have just been recently discontinued due to a lack of significant results compared to the placebo group [151]. Nevertheless, a current preclinical analysis presented pharmacokinetic and pharmacodynamic characterizations of narsoplimab. It binds to the active and zymogenic form of MASP-2 at picomolar avidity and selectively inhibits the LP without any effect on the CP or AP in ELISA experiments. These findings predestinate narsoplimab to become a candidate in clinical trials for hematopoietic stem cell transplantation-associated thrombotic microangiopathy and other LP-mediated disorders [192].

Another promising way to attenuate LP activity exclusively is the administration of specific small protein inhibitors. Szakács et al. developed a specific and highly selective MASP-2 inhibitor from the second Kunitz domain of human tissue factor pathway inhibitor-1 via in vitro evolution [168]. TFMI-2 comprises 60 amino acids and efficiently inhibits LP activity both in human and rat serum with IC_50_~ 1 µM. The inhibitor did not affect the activities of either of the other two complement pathways or the coagulation cascade. TFMI-2 variants seem to be promising candidates for further development toward the treatment of various kinds of IRI.

Concerning PRMs, there are a few examples that attempt to influence LP activity. L-fucose administrated locally at the site of damaged renal tubule cells blocked CL-K1 binding and attenuated the activation of the LP [169]. Supraphysiological concentrations of L-fucose was not toxic in mice and prevented renal ischemic injury to a large extent. Using high-dose L-fucose can be relevant to avoid the risk of renal transplant IRI. Targeting MBL could be another exciting way to inhibit LP activation. Monoclonal anti-MBL-A antibody decreased infarct size, C3 deposition, neutrophil accumulation and ICAM expression after myocardial IR in a rat model [177]. Very similar conclusions were drawn in a type 2 diabetic rat model comparing the effect of this MAb to the broad-spectrum inhibitor, nafamostat mesilate [160]. Pavlov et al. developed a new mouse model that expressed functional human MBL2. The effect of human monoclonal anti-MBL was very promising regarding all aspects of myocardial IR inhibition [178].

MBL replacement therapy aims at exactly the opposite. Instead of mitigating, LP activity is desired to be boosted in MBL-deficient individuals. Preliminary data suggested that although clinical effects were only moderate, MBL-replacement therapy could be still considered in some cases. In neonates or patients under immunosuppressive therapy, the immature or suppressed adaptive immunity carries the serious risk of severe infections. The treatment with MBL could enhance the innate immune response through lectin pathway activation [232].

### 9.2. Measurement of the Activity of Lectin Pathway

Testing complement activity is crucial in both the diagnosis of complement-related diseases and the therapeutic development of complement inhibitors. In clinical practice, if a suspicion of complement deficiency arises, first of all, the overall function of the classical and alternative pathways are tested using CH50 and AH50 assays, respectively [233]. These assays are available from many companies and applied routinely in clinics. Originally, they were based only on erythrocyte hemolysis caused by complement activity, while nowadays, they can be cell-free and fully automated. The standard methods that measure the activation of the LP are ELISA assays where the wells are coated with different activators. An ELISA kit using mannan-coated plates is commercially available for measuring the LP through MBL binding, detecting deposited C5b-9 [234]. There is no interference with the other two pathways since the effect of the AP is excluded by dilution, while the contribution of the CP is blocked by anti-C1q antibodies. The assay is robust, easy to use and can be automated. However, the mannan-coated surface applied in the kit neglects PRMs other than MBL and collectin-11. Several in-house assays are used to assess the activation of the LP via ficolins, e.g., on N-acetylglucosamine-BSA or on acetyl-BSA [235,236]. Inoshita et al. applied GlcNAc-pentamer-conjugated dipalmitoylphosphatidylethanolamine (GN5-DPPE) as an activator of the LP. MBL and ficolin-2 can bind to GN5-DPPE and initiate LP activation detected through C4-deposition [237]. To summarize, currently, there is no method that measures the activation of the lectin pathway via all PRMs simultaneously, but there are methods for the measurement of LP activation through various subsets of PRMs.

## 10. Non-Canonical Functions of the Lectin Pathway

### 10.1. Role in Embryonal and Brain Development

**Figure 6:**
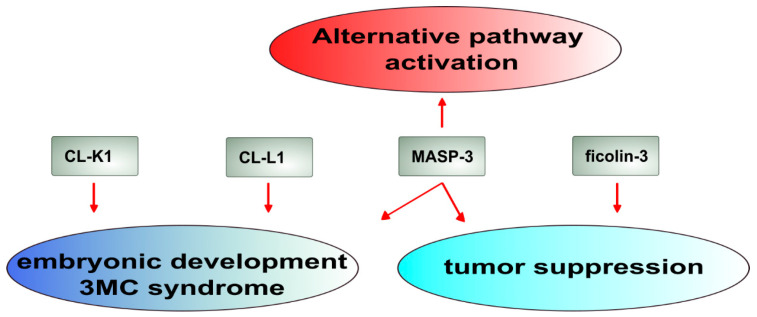
Non-canonical functions of the LP components. The components of the LP, besides their canonical functions in the complement activation, have various non-canonical functions. From this point of view, MASP-3 has a distinguished role, since it participates in embryonic development, and it has a tumor-suppressor effect as well. Red arrows point from the LP components to the observed phenomenon.

The lectin pathway has been regarded as an important immunological effector machinery in the defense against invading pathogens. It was therefore a big surprise when it was revealed in the early 2010s that this system also performs other functions beyond the complement activation [238,239]. 3MC syndrome is a rare autosomal recessive disorder which includes overlapping features of four syndromes (Maulpech, Michels, Mingarelli, Carnevale) that were considered to be separate entities previously. The characteristic features of the disease include craniofacial dysmorphism and multiple anomalies. Dysmorphic features include hypertelorism, blepharophimosis, blepharoptosis, highly arched eyebrows, and cleft lip/palate. A variable degree of postnatal growth retardation, genital anomalies, skeletal manifestations, intellectual disability, and hearing loss were also reported. Exome sequencing revealed that mutations in the *MASP1*, *COLEC10* and *COCEL11* genes are the causative agents of the disease [47]. All these genes encode components of the lectin pathway. Among the mutations described so far, the MASP-3 mutations are the most common (17 different mutations), followed by CL11/CL-K1 (11 mutations) and CL10/CL-L1 (3 mutations). Most of the mutations of the *MASP1* gene concentrate on the serine protease domain of MASP-3, resulting in the proteolytic inactivity of the enzyme [240]. As we have seen earlier, MASP-3 has an extremely narrow substrate specificity. In human blood, it has a single substrate, pro-FD [80]. Deficiency of FD, however, does not cause 3MC syndrome. There must be another substrate (or substrates) of MASP-3 outside the complement system that is cleaved during the craniofacial development in the embryonic period. It was shown earlier that MASP-3 cleaves the insulin-like growth factor binding protein (IGFBP-5), and therefore, it may liberate insulin growth factor during craniofacial development [241]. It has been reported that C1s also selectively cleaves IGFBP-5, but a deficiency of C1s does not result in developmental disorder at all [242]. Another intriguing observation is that MASP-3 KO mice do not show the symptoms of 3MC syndrome. The only difference between the wild-type and the MASP-3 KO mice is that the latter have reduced body weight by about 20% [56,57]. In zebrafish, however, gene silencing elicits the craniofacial abnormalities [239]. CL-K1 (collectin-11) and CL-L1 (collectin-10) are PRMs of the lectin pathway. Obviously, these collectins are also important in the embryonic development. They may ensure that MASP-3 cleaves its substrate in the correct place. This may explain why MASP-3 is part of the PRM-MASPs complexes, even though it is not required either for its activation or for the cleavage of its substrate (pro-FD) in the blood [243]. The selection pressure for this is obviously related to the role of MASP-3 in embryonic development (Figure 6).

Based on zebrafish *COLEC11* and *MASP1* gene knockdown experiments, the authors came to the conclusion that these proteins (CL-K1 and MASP-3) are involved in the correct migration of neural crest cells (NCCs) during embryogenesis. The *COLEC11* gene is expressed along the migratory path of NCCs in zebrafish. CL-K1 and very probably CL-L1 may serve as a guidance cue for NCC migration [239]. Another study also supported the role of the lectin pathway components in brain development [221]. However, it seems that, in this case, the normal cascade-like operation of the complement system played a role. The authors showed that the lectin pathway-mediated C3 cleavage is required for proper migration of neurons in the developing mouse brain. Deleting either MASP-1, MASP-2 or C3 in the mice resulted in the impairment of neuronal migration. In the wild-type animals, immunostaining demonstrated the presence of MASP-1, MASP-2 and C3 in the embryonic brain sections. Moreover, the activation of the complement C3a and C5a receptors rescued the migration defects. The lectin pathway appears to be involved in embryonic development in a complex and species-specific manner. In mice, the canonical function of the lectin pathway seems to be important, while in the zebrafish and humans, the non-canonical (non-immunological) function of MASP-3 plays a decisive role. Further research is needed to clarify this interesting phenomenon.

### 10.2. Role in Cancer

The role of the complement system in cancer development and progression is an intensively studied subject. The complement system plays a two-faced role in carcinogenesis: it can have tumor-promoting or tumor-suppressor activity [244,245]. In this regard, MASP-3 is a highly interesting protease. Several studies reported mutations in the *MASP1* gene in colorectal and breast cancer [246,247,248]. One study showed that the expression of MASP-3 is significantly (up to a thousand-fold) downregulated in colorectal tumor tissue compared to normal mucosa [247]. Immunohistochemical analysis also supported this notion: MASP-3 could not be detected in the tumor tissue while it was present in the corresponding normal mucosa. Moreover, overexpression of MASP-3 significantly reduced cancer cell proliferation in vitro compared to the control cells. Finally, in a mouse xenograft model, overexpression of MASP-3 in a colorectal cancer cell line (HCT116) prevented the tumor formation when injected subcutaneously in the nude mice, while the control cells yielded fast-growing tumors. Conversely, gene silencing had the opposite effect: it resulted in a significant increase in tumor growth compared to the control cells. All these results prove that MASP-3 is a tumor-suppressor protease. This is very interesting because most of the proteases studied in various cancer tissues play a tumor-promoting role from cell transformation to tumor invasion and metastasis. They can cleave the extracellular matrix facilitating tumor invasion and metastasis or process and activate specific cytokines and growth factors necessary for tumor growth and angiogenesis. There are only a few examples for tumor suppressor proteases [249]. MASP-3 belongs to this special group of proteases. As in the case of the 3MC syndrome, we do not know what substrate (or substrates) is cleaved by MASP-3 in the tumors which is necessary to mediate the tumor-suppressor effect.

Interestingly, ficolin-3, to which MASP-3 is predominantly associated in human serum [41], also may act as a tumor-suppressor agent. It has been shown that the expression of the *FCN3* gene is lower in the case of different cancers [250], and a recent study demonstrated that hepatocellular carcinoma patients with a high *FCN3* expression had a significantly better overall survival [251]. Although the mechanism is far from clear [252], we can speculate whether MASP-3 may contribute to the tumor suppressor phenomenon in this case.

Complement activation can promote tumorigenesis via diverse mechanisms. The generation of C3a and C5a can suppress the adaptive immune response against cancer cells. The mechanism through which C3a and C5a is generated can be different in the different tumors. Recently, it has been reported that the lectin pathway plays a decisive role in pancreatic ductal adenocarcinoma (PDA) [253]. The authors showed that fungi can migrate from the gut lumen to the pancreas and that the pathogenic mycobiome can trigger lectin pathway activation. The mycobiome of PDA tumors is rich for *Malassezia* species in both mice and humans. *Malassezia* binds MBL and initiates the lectin pathway promoting tumorigenesis. In MBL-null mice, *Malassezia* did not accelerate tumor progression. The cleavage of C3 is also essential, since C3 knockout mice were protected against PDA progression. The knockdown of C3aR in tumor cells also mitigated tumor growth. Although the authors did not investigate this, it is obvious that MASPs play an indispensable role in the oncogenic progression by cleaving C4 and C2, the components of the convertase complex. Preventing lectin pathway activation by inhibiting the MASPs in PDA may be a therapeutic option.

## 11. Summary/Perspectives

Since its discovery at the end of the 20th century, our knowledge about the LP has been changed considerably. It turned out that besides being the third activation route of the complement system, it has many connections to other physiological systems and has a diverse function in the body. In the strict sense, the LP is an activation pathway enzymatically very similar to the CP, but it does not require antibodies to recognize the dangerous structures. The PRMs independently bind to PAMPs and DAMPs and initiate the activation of the complement cascade. A crucial turning point in LP research was the realization that the LP is extremely closely related to the AP. MASP-3 is considered a member of the LP, since it circulates in a complex with the PRMs. However, we can rightfully consider it to be part of the AP as well, since it is solely responsible for activation of pro-FD in the blood. MASP-1, the most abundant protease component of the lectin pathway, also influences AP activation, although the mechanism is not clarified yet. The exploration of the fine details of the interaction between the two activation pathways requires further research. The LP is the only complement activation pathway whose protease components have been shown to directly activate cells through the cleavage of protease-activated receptors. In this respect, MASP-1 plays a major role in activating different cell types and different signal transduction pathways. The importance of direct cell-activation by LP components is further highlighted by its evolutionary conservation, which raises the need for further research on other cell types (particularly on various leukocytes) to attain a more comprehensive understanding of the cellular effects of LP serine proteases. The discovery of the role played by LP components in embryonic development surprised the entire research community. Consequently, MASP-3, as a physiologically multifunctional protease, has received considerable attention. Unfortunately, the mechanism behind this phenomenon is totally unknown. The same is true for the tumor-suppressor effect of MASP-3. The research of these moonlighting functions of MASP-3 is of great importance. The crosstalk between the lectin and the coagulation pathway is also an interesting research area. New experimental models, including the application of whole-blood system and surfaces covered with endothelial cells can yield novel results in the near future. The most important application of the results achieved in the research of the activation and regulation of the complement system is the development of drugs to treat complement-related diseases. In this respect, the LP is somewhat behind the other two pathways, since there is no FDA-approved drug at the moment, which would act exclusively on an LP component. MASP-2 is a very attractive target, since it is an essential component of LP activation, and it has a low serum concentration. Experiments with MASP-2 inhibitors in different types of IRI provided promising results, and several pharmaceutical companies keep MASP-2 inhibitors (monoclonal antibodies, peptides, small molecules) in their pipeline. MASP-1 and MASP-3 also can be pharmaceutical targets due to their various physiological roles. It is predicted that the first LP-related drugs will be approved in the near future by the authorities.

## Figures and Tables

**Table 1:** Lectin pathway components involved in human diseases and LP-related drugs in clinical or preclinical trials.

Disorders	LP Components Involved	Drugs under Clinical Trial	Drug Candidates under Preclinical Trial
**Renal disorders**			
IgA nephropathy	MBL, ficolin-2, MASP-1, MASP-2, MASP-3, MAP-19 [145,146,147,148,149]	Narsoplimab (recently discontinued) [150,151]	Anti-MASP-3 (OMS906) [152]
Membranous nephropathy	MBL, MASP-1, MASP-2 [153,154,155,156]	Narsoplimab [157]	
Diabetic kidney disease	MBL, ficolin-3 [158,159]		Anti-MBL MAb [160]
**Ischemia Reperfusion Injury**			
Renal IRI	MBL, CL-K1, MASP-2 [76,77,78,161,162,163,164,165]		CL-K1 inhibition by L-fucose; C1INH; TFMI-2 [166,167,168,169]
Myocardial IRI	MBL, ficolin-2, MASP-1, MASP-2 [138,170,171,172,173,174,175,176]		C1INH; anti-MBL MAb [177,178]
Ischemic stroke	MBL, MASP-1, MASP-2 [179,180,181,182]		
**Atherosclerosis**	MBL, ficolin-1, ficolin-2, ficolin-3, MASP-3 [183,184,185]		
**COVID-19**	MBL, ficolin-2, ficolin-3, MASP-2 [186,187,188,189,190,191]	Narsoplimab * [192]	
**Hereditary angioedema**	MASP-1 [193,194]	C1INH (approved) ** [195]	
**Schizophrenia**	MBL, ficolin-2, MASP-2 [59,196]		

* Target: MASP-2; ** Main targets: kallikrein and FXIIa.
